# Supplementary material for: Allele-level visualization of transcription and chromatin by high-throughput imaging
Source: Histochem Cell Biol. 2024 May 9;162(1-2):65–77. doi: 10.1007/s00418-024-02289-7 (PMC11227451; doi:10.1007/s00418-024-02289-7)
Supplement: Supplementary file 1 — Supplementary file1 (PDF 603 KB) [file 418_2024_2289_MOESM1_ESM.pdf]

Supplementary Information

Supplementary Figure 1: Sequence and location of DNA and RNA probes binding sites

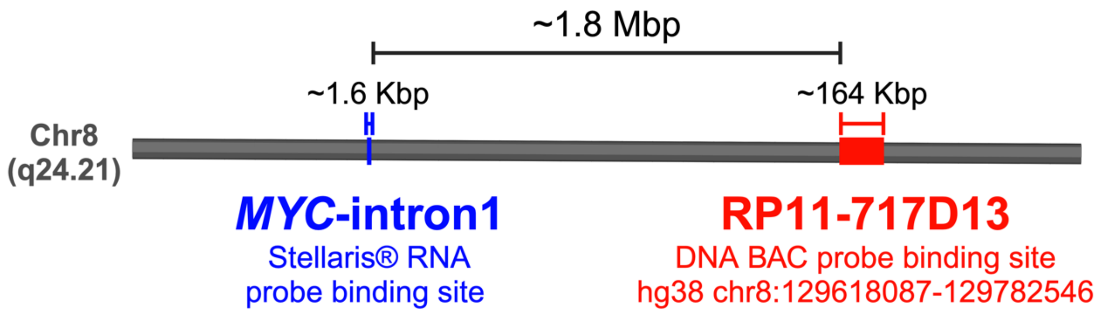

MYC Stellaris® RNA Probes hybridization sites

(Intron1, hg38 chr8:127736231-127737854, strand: +, 5' to 3')

gtaagcaccgaagtccacttgccttttaatttattttttatcacttt**aatgctgagatgagtcgaat**gcctaaatagggtgtcttt
tctcccattcctgcgctattgacacttt**tctcagagtagttatggtaa**ctggggctggggtgggggtaatccagaactggatc
g**gggtaaagtgacttgtcaag**atgggagaggagaaggcagagggaaa**acgggaatggtttttaagac**taccctttcgaga
tttctgccttatgaatatattcacgctgactcccggcc**ggtcggacattcctgcttta**ttgtgtt**aattgctctctgggttttgg**g
gggctgggggttgctttgcggtgggcagaaagccc**cttgcatcctgagctccttg**gagtagggaccg**catatcgcctgtgtg**
agccagatcgctccgcagccg**ctgacttgtccccgtctccg**ggagggcatttaaatttcggctcaccgcatttctgacagc
cggagacggacactgcggcgcgtcccgcccgcctgtccccgcggcgattccaa**cccgccctgatccttttaag**aagttgg
catttggctttttaaaaagcaataatacaatttaaaacctgggtctctaga**ggtgttaggacgtggtgttg**ggtaggcgcaggc
aggggaaaagggaggcga**ggatgtgtccgattctcctg**gaat**cgttgacttggaaaaaccag**ggcgaatctccgcaccc
agccctgactcccctgccgcggccgccctcgggtgtcctcgcgcccgagatgcggaggaactgcgaggagcggggctctg
ggcggttc**cagaacagctgctacccttg**gtggggtggctccgggggaggtatcgcagcggggtctctggcgcagt**tgcatct**
ccgtattgagtgcgaag**ggaggtgcccctattattat**ttgac**accccccttgtatttatgga**ggggtgttaaagcccgcggc
tgagctcgccactccagccggcgagagaaagaagaaaagctggcaaaaggagtgttggacgggggcggtactgggggtgg
ggacgggggcggtggagagggaaggttgggaggggctgcggtgccggcgggggtaggagagcggctagggcgcgagtggga
acagccgcagcggaggggccccggcgcggagcggggttcacgcagccgctagcgcccaggcgcctctcgccttctccttc
aggtggc**gcaaaactttgtgccttgga**tttt**ggcaaattgttttcctcacc**gc**cacctcccgcggcttcttaa**gggcgcca
g**ggccgatttcgattcctctg**ccgctgcggggccgactcccgggctttgcgctccgggctcccgggggagcgggggctcgg
cgggcaccaagccgctggttca**ctaagtgcgtctccgagata**gcaggggactgtccaaagggggtg**aaagggtgctccc**
tttattccccccaccaagaccacccagccg**ctttaggggatagctctgca**aggggagaggttcgggactgtggcgcgcact
gcgcgctgcgccaggtttccg**caccaagacccctttaactc**aa**gactgcctcccgctttgtgt**gccccgctccag

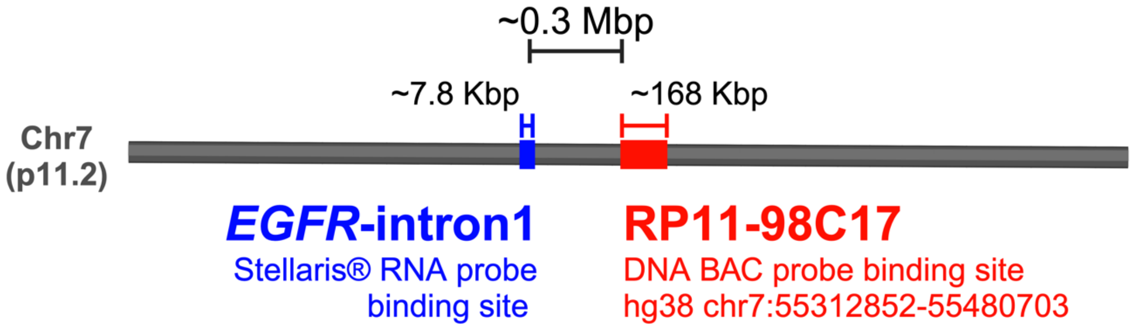

EGFR Stellaris® RNA Probes hybridization sites

(the initial 7,843 nucleotides of Intron1, hg38 chr7:55019366-55027209, strand: +, 5' to 3')

gtaagggcgtgtctcgccggctcccgcgccgcccccggatcgcgccccggaccccgcagcccgcccaaccgcgcaccggcgcaccggctc
ggcgcccgcgcccccgcccgtcctttcctgtttccttgagatcagctgcgccgccgaccgggaccgcgggaggaacgg**gacgtttcgttcttcg**
gccgggagagtctggggcgggcggaggaggagacgcgtgggacaccgggctgcaggccaggcggggaacggccgccgggacctccggcgc
cccgaaccgctcccaactttcttccctcactttccccgcccagctgcgcaggatcggcgtcagtgggcgaaagccgggtgctggtgggcgcctg
gggccggggtcccgcacgtgcgccccgcgctgtcttcccagggcgcgacggggtcctggcgcgcacccgaggggcgggcgctgcccacccgc
cgagactgcactgtttagggaagctgaggaaggaacccaaaaatacagcctcccctcggaccccgcgggacaggcggctttctgagaggacct
ccccgcctccgccctccgcgcaggtctcaaactgaagccggcgcccgccagcctggccccggcccctctccaggtccccgcgatcctcgttc
cccagtgtggagtcgcagcctcgacctgggagctgggagaactcgtctaccacctgcggctcccggggaggggtggtgctgg**cggcggtta**
gtttcctcgttggcaaaaggcaggtggggtccgacccgccccttgggcgcagaccccggccgctcgcctcgcccggtgcgc**cctcgtcttgcc**
tatccaagagtgcccccacctcccggggaccccagctccctcctgggcgcccgcgccgaaagccccaggctctccttcgatggccgcctcg
cggagacgtccgggtctgctccacctgcagcccttcggtcgcgcctgggcttcgcggtggagcgggacgcggctgtccggccactgcaggggg
gatcgcgggactcttgagcggaagccccggaagcagagctcatcctggccaacaccatggtgtttcaaaatggggctcacagcaaacttctcct
caaaacccggagactttctttcttggatgtctcttttgctgtttgaagaatttgagccaaccaaaatattaaacctgtcttacacacacacacacac
acacacacacacacacaccggattgctgtccctggttcaagtgtgccaagtgtgcagacagaacatgagcgagtctggcttcgtgactacc**gac**
cataaacccacttgacagggga**aacatgccttggaaggttta**attgcacaattccaaccttgagctgcgcgggttccaagagccaggcccgta
ct**tgctgttgatgtcattggct**tggggagttggggtttggtgcccagcgcggtcgttgggggaggggcaaggcatagaacagtggttcccagacctt
gctgcacattggaattacctgggattaaaaaaaaaaaatcaaaacaaaaaccagtgtctggctcccgcccccagacattctgatttaattggca
tggggcaagacctggacttgggatttttttaatgctcttcatgtgatctgttgggcagccagatttggggatcactagacggaagaaggattgttaaag
tctccggagatgttacttgccaatgctaagagctct**ttgaggacatctggaattgt**tacaatattgccaaatataggaaagagggaaaaggtagag
tgtgattccaataataaaggattccgcttttcattgaaggaac**tggtggaaaggtttcttctc**tgctgagcctgcaggcccgtcctgcctgcctggg
gtgcccgggagacgcgggcctgctccggagactgctgactgccgg**tcctgttagtcaggtgtcag**ccctgtctctgccgaagagactcttctcttt
attttaaattaaaccctcagagcaccaccaaagcatcacttttctccctccattggtgttctcattctttgatgttacttgtttgaacaccactattagt
agttggagatttgttcctgagaaaaatataaataccacttaatttgcctgtttgtcccgcatt**cactcaaaacagaatgctcc**tgaagacaagaga
gagagtaggagaacagacgctattccattacagtaacataaaagact**ggattttcaggggcaaatta**ttaaaataggagatgagctcttttaaca
gaaatttgtttaaggcctgtgtctatcaaattcagtggattttattcaagatgcactttgtttagtgggagttttgtttg**gttctgggacatgctaactt**cta
g**acttgctgctcttagaggta**atgactgccagacaccatttcatgagtcctaatccccacattaagcataagaggtgcacactctcctcctatgg
gggaaactgaggtacgaagaactaaagtgactttcccacagctggtgggaggcagacgggaaattcacaccaggggcttccaactccagatcc
ctctctcaacttccaaactccactgccttgtccgagttctggtttcaggagatcc**aaatcaggtgtgtgcaaatg**tctaatgtcagagctggcaag
gggaaagggcccagggagccggctcatgacgatgagcctgtctgaagcttcaacgcgggctgtccggcagtctgcattcctgcc**gagttcctca**
gccctctgttgggtcaccttccatagaggcagcttagtcctcagttcagtgagcatggagtggagactgcttgaggggtgctgagcaaagccctgc
ctcttacaggatgaaggtgctctccagaagggacactggaaagtattccaaggc**gagtcgaattcccaactgag**ggagctttgtggaaataagc

ccgcccagccccacttct**ggagacgttcccattcagta**gg**tccgagctgtcttaaagaga**aaccaaagtggggatattaatggtatccaaagt
gagatctaccccaccctccctcctcaaaggaggtcagatcaagaaagcccaagcccggcctggcaattgggacctttcttctcactccagccc
agggtgaaggtggacaagtc**actttgacccttcaggcttc**tgagctgttgtttctgaattcagtgaatatttactgagtgcatagaatatgctagatat
tctgggctaaaggttgaaggggggggtgagttt**taagggtttctgctcttgct**tccagattgctttcaaatctggaaaggacaccagtggtttgtgtgtta
gacccacactgccgtag**cacagaatacaagaaactgg**ctgagagctccaataggcttttaacagtaatttctggcttcacgtatttagtttcataa
ctcatgatttttcaaaaacttctggtttgaagaca**ccgattgccgaaagtccatt**gtgctgcataattacacttggtccacgtgacagcactaacat
gttctgaaatgtttttagaagtagtctcagcaaagatgaaggattcctccctgtttgaaaagaaaatattctttgttttttctttgatctaagctctaagac
tagcagctagcatctgaaactttttgacg**agagtgacaaaccaactcta**atattaaag**gcaattgatgattatgggca**ctgaagggaaggtaa
ccccaggctggtgccccggaatagggatgggtcacaat**gttgaggacatttcgcctgt**tgcagaacccacctgcaacacagtgtggcccttgcc
atgtgacttgtgtgtgtgcctgtgtgtctgtgtgtgcgtgttttaattttgacttcataagtactctagttatgagcttatttaacattgggttttactaatagg
ggtatgtgttgagaaaatttcaaagttttagaatat**ggttcacccacatgttgctt**ccctgtaaatataatttttaaaaccagattctgggccgggcat
ggtggctcacctctataatcccaaaacgttgggaggccgaggcaggcgaatcatgaagccaggagtttgagaccaggctgaccaacacggtga
aacccagtctctactaaaaatacaaaaaaaaattagctgggcgtggtggcaggtgcctgtaatcccagctactcaggaggctgaggcaggagaat
tgcttgaacccaggaggcagaggttgcagtgagccaagatcgcaccattgcactccagcccgcgcgacagtgtgagactccatctcaaaaaaa
aaaaaaaaaaaacagattctgttcctcagatccattccatttttgttttcctttatcacttatggacatttgaaattatggtaataaacattgttagtctc
agttaattattactggtttattcttgaaccactaatccatagagaatagagtgtaaatctt**aacttgttcctgtaggccat**ccccattaaacatcata
gtgttttctcattcgttctttttcgttttcctcctacaggaatgaattttctaagaaaattccagcagttggctctt**tggacgacatctctagattg**tcctc
cattgggcccataggcacaagctggccagt**ttgaatttgggcaagaatcc**aggcattggaacttattcaaataactagtttgcctgtaattttcact
ttttcagagtcatctgataaagctttcttgctacacatttagatagatacactcaatccagttgtctagaaagttccctgagccagctgggagcagga
ggggtagttggggccaggaatattgggggt**gtgtttactgagcccctaga**aagtaagtgctagatttgacatttcaatccctgaaggccctgaagtt
cagtatcaaatgactggtcctgtggactgagcatctgtgaattgcatatgcttagagtaaatttt**actcctaccagtttcagcag**cttgctttagcaa
gcagtatggaaacactaacatgggggagtagaatttctctctctgatccaagttttatctcattctggtgggttttcaaggagagactcggagtccaa
gtgtcctttctgaatatatctggaacttctcattaacaaaagactcaagttataatttaggggacaaggcacccaatgagaatgccttgcaggcagc
cctaagtacacctgcaattacaccattactagcgcggcagcac**acatggccctgacttagttt**aaataattacgtaagtcaaccatgattgtttg
ccctttgcatagaagggcaagtattggtacctgttacaacttaggcttttttttctttatgtttgagccatgatgagtgatttacactgttgcatccatatgt
tgagatgtaagaataaattagacttggta**attgcccttaagtgtctgga**agtcaactggggaaagagagctagagataataagtgtgaaacaatgt
cacagaatcaatgacggaactcttccc**aggacaaaggatgacttttg**agttcagtcttt**gcctttaattctacatgggg**aggagagc**acgtttag**
ccacaaatggaagggattactcatttgagctatttggttatatgattatttccccagagaataggatgtgcagggcattacacaagcagtgccaata
gcagcaaagttcttgagagtgctagtaattcaaatggcaggaagagaaggaataaatggtaaggctacctacagttcacagagagctccatcctc
actgtggctttggattttgtcctgtgtgaaagagaagtgactgtgaactgacatgctgtgtttggtgttttagaaagatggctgcagcagcggtttgggg
aatggactgcaggagtggcattggaaacaggaaggttcatgactattgccagagacagaggatgaagcaggagcaaggaagattcaggacagg
ggactccggggctgatcaggaggcagaactggttgataagtatatgtagcagcataagaaagaaagaatcccagattgacacccaggcttctca
cttggaagcctggatagatactgaatgcaatcacaaaggctgggaagtcaatgggactgcagggaagggaagggaagggaggagaagaggaag
ggcaggagggtccaatatcaatattcagcttttagatgtgttgagcttgaagtgctcagatggagaagtccaggaggcagtagaatacggtggtcca
gagcacaggagagcaatgtggcttgagttgtcatttgctcacatatttccgtgtcagttacttgtcttagatcacagaacaagttctc**ctctcacagtt**
tcctggctccacctgtctcatgctc**accgtcagcatcgaaattga**gccacaccaggggtt**ctggataccagcttctctct**aggtgaggctgcta
tag**tcagcagctgattagttgca**gttatcagcaactggtaatataatatattgtgcatataagtgtaccagaagtcatgtttata**tattgctgcaaat**
actcggaatggggatctctt**gttccctgcttaagaccaca**tcacattacttggttttgtacgctagtggctgaaccaaaaaaaagtaggagatgattt
tttttctttttcttaaagcagtagcttttgaaccttgaccatgctttctaaccagctgaggggcttttgaaaaagagggtgccttactgtgccccagac
caggacaatcagtatttctggggaatggagcctggcacacacacatttcttaaagctcccttggcaattc**tgaggagtggattacatgtt**gtatgt**a**
gctcgtaacgaaagaaatcttgtc**tttgctctcagacccccatt**tcttactcatctcatgagct**ccttcgagatccagaaacag**ttgcatatttc

attagtaaatcagttccagagtcacattttatttcacaagttagtccattaaaagtttcctgcagtgaggaaatagccagaaagaacactccaccc
ctcctccttttataactatagggtctggctcgacagagcaggagcatcgccatcttggacaagcccctcattctaaagttcaccttaataaaaaac
tgcctaaattcaaactgcatcagcctaatggctaaggtcagcatgaccataaaccacaaataacatctccaaccggaaacattcgaaactcctc
ctcgaccagagacatgctagtcccgagataaccccctccagcagggaagatgccagtctcgggataacctctctctggccggaaagatgcct
gccccaagataaacttgcctcctcccagagatattccaaccctgccataaaacttctccctcaaacaggaacattccaaaattctgataatctcc
ctcaccctaaaaccaatatatactcctagtctgtaagagaaagcgctcttgaccaaaattcaccaggagtgcctcccaggttttaactaaagaaa
acctctctttaactgccaaaaaaaaaaagggaaaaaaaaaagctttctgcagtggctttcagcgggcccagcatggcagcagcacctgagaac
ctgttggagatgcacactcttggaccccaccctggcctctgagtaagacactggaagggcaggccccggtctgtgcacacaagtcctcagggag
attctgactgatgcatgccagattttgagaactgctgatatactccaggcacatcgcatgctgggatctagatacaccaagggaacaaaataactg
cacttgtcctctgaggaccgacttacctttggaagggctgagaaagagagacacatacaagatcactccctgt

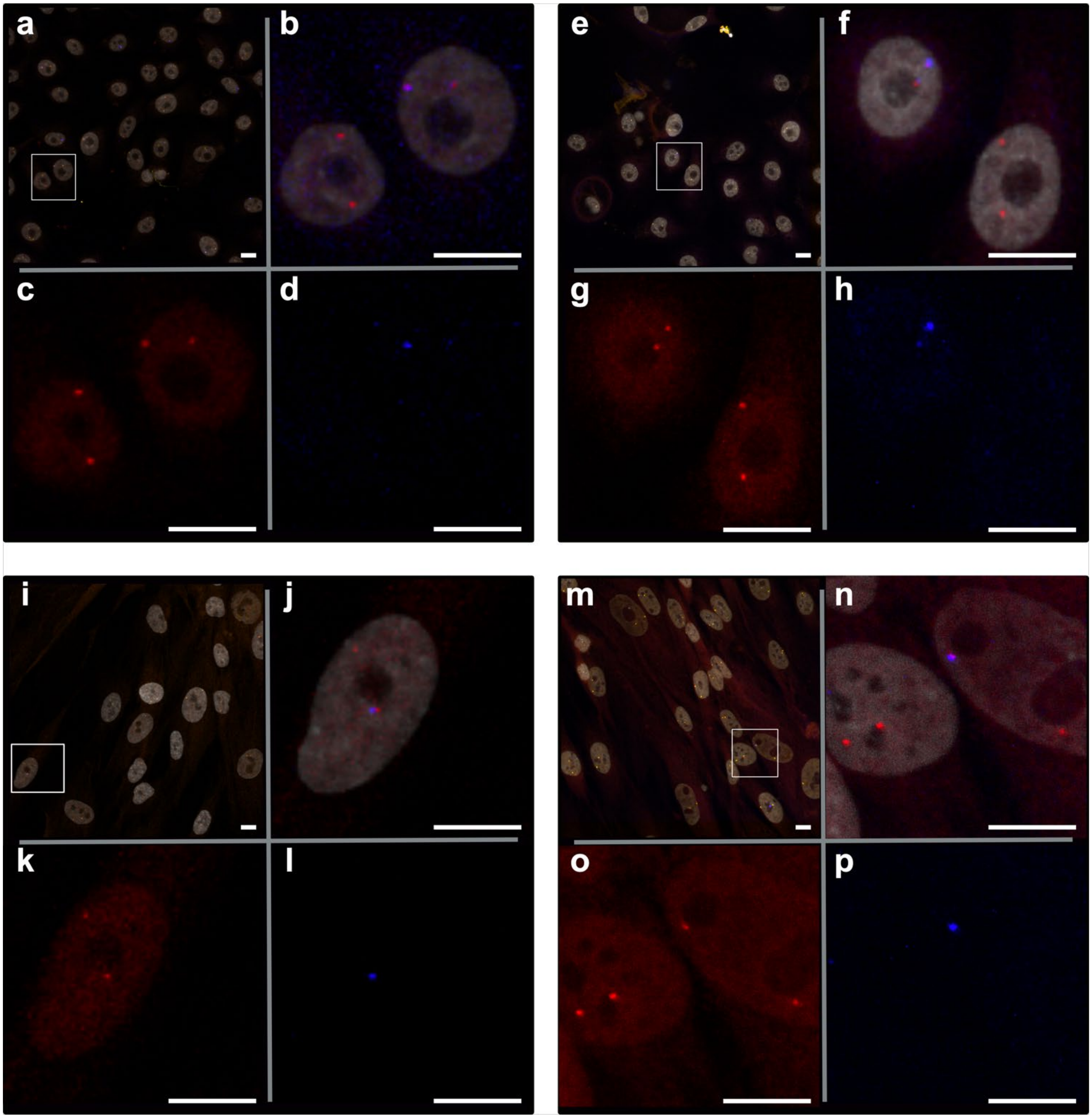

Supplementary Figure 2: Representative images of simultaneous and sequential MYC DNA/RNA HiFISH in HBEC and HFF cells. Red: DNA, blue: nascent mRNA, grey: DAPI-stained nucleus. (a-d) simultaneous FISH in HBEC. (e-h) sequential FISH in HBEC. (i-l) simultaneous FISH in HFF. (m-p) sequential FISH in HFF. Scale bars: 10 µm

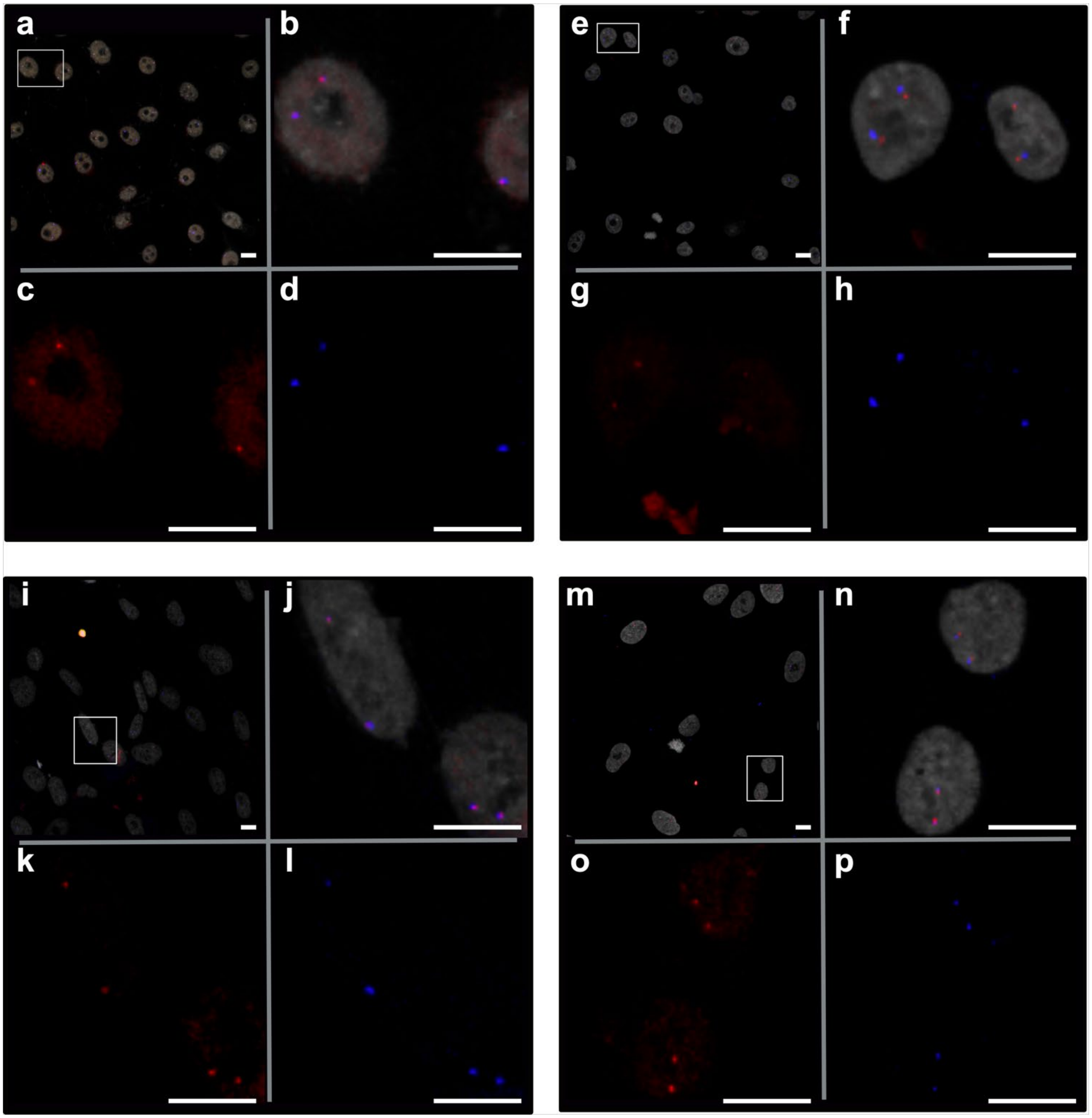

Supplementary Figure 3: Representative images of simultaneous and sequential EGFR DNA/RNA HiFISH in HBEC and HFF cells. Red: DNA, blue: nascent mRNA, grey: DAPI-stained nucleus. (a-d) simultaneous FISH in HBEC. (e-h) sequential FISH in HBEC. (i-l) simultaneous FISH in HFF. (m-p) sequential FISH in HFF. Scale bars: 10 µm

Supplementary Table 1: Summary of radial distance means, standard deviations, and KS test results comparing active to inactive alleles across all experimental repeats.

Condition					Radial distance			Two-sided Kolmogorov–Smirnov (KS) Test		
**Gene**	**Cell-line**	**Protocol**	**Repeat**	**Activity**	**N Allele**	**Mean**	**SD**	**N Allele**	**p value**	**D Statistics**
MYC	HBEC	Simultaneous	1	Active	1179	0.7	0.2	1914.00	0.37	0.04
				Inactive	735	0.7	0.2			
MYC	HBEC	Simultaneous	2	Active	125	0.6	0.2	234.00	0.33	0.12
				Inactive	109	0.7	0.2			
MYC	HBEC	Simultaneous	3	Active	1629	0.7	0.2	2978.00	0.34	0.03
				Inactive	1349	0.7	0.2			
MYC	HBEC	Sequential	1	Active	781	0.7	0.2	1380.00	0.22	0.06
				Inactive	599	0.7	0.2			
MYC	HBEC	Sequential	2	Active	154	0.7	0.2	344.00	0.42	0.10
				Inactive	190	0.8	0.2			
MYC	HFF	Simultaneous	1	Active	246	0.7	0.3	490.00	0.86	0.05
				Inactive	244	0.7	0.2			
MYC	HFF	Simultaneous	2	Active	258	0.7	0.2	652.00	0.01	0.14
				Inactive	394	0.7	0.2			
MYC	HFF	Sequential	1	Active	159	0.7	0.2	790.00	0.13	0.10
				Inactive	631	0.7	0.2			
MYC	HFF	Sequential	2	Active	73	0.7	0.2	150.00	0.28	0.16
				Inactive	77	0.8	0.2			
EGFR	HBEC	Simultaneous	1	Active	3545	0.6	0.2	4614.00	0.40	0.03
				Inactive	1069	0.6	0.2			
EGFR	HBEC	Simultaneous	2	Active	7891	0.6	0.2	9776.00	0.00	0.05
				Inactive	1885	0.6	0.2			
EGFR	HBEC	Simultaneous	3	Active	7623	0.6	0.2	9108.00	0.00	0.05
				Inactive	1485	0.6	0.2			

EGFR	HBEC	Sequential	1	Active	326	0.6	0.2	504.00	0.39	0.08
				Inactive	178	0.6	0.2			
EGFR	HBEC	Sequential	2	Active	116	0.6	0.3	166.00	0.65	0.12
				Inactive	50	0.6	0.3			
EGFR	HFF	Simultaneous	1	Active	2554	0.6	0.2	3458.00	0.74	0.03
				Inactive	904	0.6	0.2			
EGFR	HFF	Simultaneous	2	Active	1184	0.6	0.3	1902.00	0.04	0.07
				Inactive	718	0.6	0.3			
EGFR	HFF	Simultaneous	3	Active	2159	0.6	0.2	2748.00	0.14	0.05
				Inactive	589	0.6	0.2			
EGFR	HFF	Sequential	1	Active	208	0.6	0.2	368.00	0.05	0.14
				Inactive	160	0.7	0.2			

Blue font color rows indicate data corresponding to Fig. 4

Supplementary Table 2: List of change in radial distance mean and KS test comparing simultaneous vs. sequential FISH

Condition				Radial distance	Two-sided Kolmogorov–Smirnov (KS) Test		
**Gene**	**Cell-line**	**Activity**	**Protocol**	**Δ mean**	**N allele**	**P value**	**D statistics**
MYC	HBEC	Active	Simultaneous<br>Sequential	0.06	1960	0.00	0.23
MYC	HBEC	Inactive	Simultaneous<br>Sequential	0.06	1334	0.00	0.25
MYC	HFF	Active	Simultaneous<br>Sequential	0.06	1186	0.02	0.11
MYC	HFF	Inactive	Simultaneous<br>Sequential	0.04	1474	0.00	0.14
EGFR	HBEC	Active	Simultaneous<br>Sequential	0.03	3871	0.16	0.07
EGFR	HBEC	Inactive	Simultaneous<br>Sequential	0.04	1247	0.05	0.11
EGFR	HFF	Active	Simultaneous<br>Sequential	0.04	2762	0.06	0.10
EGFR	HFF	Inactive	Simultaneous<br>Sequential	0.09	1064	0.00	0.17
